# Population-based e-records to evaluate HPV triage of screen-detected atypical squamous cervical lesions in Catalonia, Spain, 2010–15

**DOI:** 10.1371/journal.pone.0207812

**Published:** 2018-11-26

**Authors:** Silvia de Sanjosé, Vanesa Rodríguez-Salés, Xavier F. Bosch, Raquel Ibañez, Laia Bruni

**Affiliations:** 1 Cancer Epidemiology Research Programme, IDIBELL, Catalan Institute of Oncology, L'Hospitalet de Llobregat, Barcelona, Spain; 2 Centro de Investigación Biomédica en Red en Epidemiologia y Salud Pública (CIBERESP), Barcelona, Spain; 3 Reproductive Health Global Program, PATH, Seattle, United States of America; 4 CIBERONC, Madrid, Spain; Universidade Estadual de Maringa, BRAZIL

## Abstract

Equivocal lesions (ASC-US) are common abnormalities in cervical cancer screening exams. HPV testing helps to stratify the risk of progression to high-grade squamous intraepithelial lesions or more (HSIL+). Population-based medical electronic data can be used to evaluate screening recommendations. The study uses routine electronic data from primary health centers to estimate the impact of HPV testing in a 3- and a 5-year risk of HSIL+ after an ASC-US. The study includes data derived from medical electronic information from 85,775 women who first attended a cervical cancer screening visit at the National Health System facilities of Catalonia, Spain, during 2010–11 and followed up to 2015. Included women were aged between 25–65 years old, having at least one follow-up visit, and a cervical cytology of ASC-US (N = 1,647). Women with a first result of low-grade squamous intraepithelial lesions (LSIL) (N = 945) or those with negative cytology (N = 83,183) were included for comparison. Those with a baseline HSIL+ were excluded. Incident HSIL+ was evaluated by means of Kaplan-Meier curves and multivariate regression models. HPV test results were available for 63.4% of women with a baseline ASC-US. Among all ASC-US, 70 incident HSIL+ were identified at 5 years. ASC-US HPV positive women had a high risk of HSIL+ compared to women with negative cytology (adjusted HR = 32.7; 95% CI: 23.6–45.2) and a similar risk to women with baseline LSIL (HR = 29.3; 95% CI: 22.4–38.2), whereas ASC-US HPV negative women had no differential risk to that observed in baseline negative cytology. Women with ASC-US and no HPV test had an average HSIL+ risk (HR = 14.8; 95% CI: 9.7–22.5). Population-based e-medical records derived from primary health care centers allowed monitoring of screening recommendations, providing robust estimates for the study outcomes. This analysis confirms that HPV testing improved risk stratification of ASC-US lesions. The information can be used to improve diagnosis and management of screen detected lesions.

## Introduction

Equivocal lesions (atypical squamous cells of undetermined significance [ASC-US]) are common abnormalities detected in population-based cervical cancer screening [1,2]. However, not all women with a cytology diagnosis of ASC-US have the same risk to develop or harbor high-grade squamous intraepithelial lesions or more (HSIL+), and triage with oncogenic or so-called “high risk” human papillomavirus (HPV) types through HPV DNA detection is now considered essential to determine risk and subsequent management [3–5]. More than half of ASC-US lesions are estimated to harbor HPV DNA, providing a higher accuracy to detect cervical intraepithelial neoplasia grade 2 or more (CIN2+) lesions as compared to repeated cytology [6]. ASC-US HPV positive women have been showed to have a cumulative risk of CIN2+ that goes beyond 10% and above 4% of CIN3+ in long follow-up series [1,7]. ASC-US HPV positive women therefore need close follow-up until a repeated negative cytology or a negative HPV test exclude ongoing disease [4,8]. Conversely, ASC-US HPV negative women have a very low risk of CIN2+ and are recommended to follow a regular screening interval [1,9].

In Spain, a country with historically low rates of invasive cervical cancer, screening has largely been based on conventional cytology under an opportunistic scenario. Systematic evaluation of screening records in the region of Catalonia indicate that abnormal cytology results account for around 3% of cervical cytologies in any given screening round, of which 96.5% are ASC-US or LSIL [2]. At present, HPV testing is widespread for ASC-US management [10,11]. This study explores the incidence of HSIL+ among women attending their first cervical cancer screening exam in a minimal window of 3 years to follow-up.

## Material and methods

This study was approved by the Clinical Research Ethics Committee and the Institutional Review Board of the University Institute for Primary Care Research (IDIAP) Jordi Gol (P15/106).

In the region of Catalonia, opportunistic cervical cancer screening is offered for free by the National Health System (NHS) and delivered through the public primary care network. Since 2006, Catalan NHS guidelines established that a 13 high-risk HPV types DNA test (HC2, Qiagen, Germantown, MD) was recommended after a diagnosis of ASC-US within 3 months of the conventional cytology result. Women with an HPV negative test are sent back to a 3-years regular screening schedule while those with an HPV positive result are referred to immediate colposcopy [12].

Specific information for the purpose of this analysis was extracted from the NHS electronic data provided by the Information System of the Primary Health Care facilities (SISAP). The SISAP holds data from 279 Primary Health Centers and 28 Sexual and Reproductive Health Clinics. The SISAP stores the NHS primary care clinical data of approximately 75% of the resident population in Catalonia [13,14]. We requested information on age, place of birth, recruitment health region, date and results of cervical cytology and HPV testing, and ICD-10 codes for any cervical-related intraepithelial lesion or cancer [15]. Age at first cytology was estimated as the number of complete years between July 1 in the woman’s year of birth and the date of the first cytology. Cytology refers to conventional cytology evaluated at the local pathology departments. The results were reported according to the Bethesda System categories as carcinoma, HSIL, atypical squamous cells cannot rule out HSIL (ASC-H), LSIL, ASC-US, and negative for intraepithelial lesion or malignancy [16]. The outcome selected for this analysis was HSIL+ that included cytology results of HSIL and cervical carcinoma or histology (when available) diagnosis of CIN2, CIN3 or cervical squamous carcinoma, cervical adenocarcinoma, and cervical adenosquamous.

The HPV DNA test result was included in the analysis if it was performed within a window of 60 days since the first cytology record. A window of 90 days would have resulted in including additional 62 women of which 52% remained positive. All women with a baseline diagnosis of ASC-H and HSIL+ were excluded from this analysis as these women are referred for immediate colposcopy and treatment.

### Study population

We identified 142,284 women aged 25–65 residing in Catalonia and having a first cervical cancer screening visit during 2010–11 at NHS facilities. Women with a baseline diagnosis of ASC-H or HSIL+ (N = 453) were excluded from this analysis, leaving 141,831 women as the target population. Of them, 85,775 women (60.5%) were included in the final analysis as they had at least one additional screening test up to December 2015 when we closed the study window time. The remaining women (N = 56,056, 39.5%) were considered lost to follow-up as they did not return within an interval of 3 years or more after the first cytology. These women were significantly older (44.3 vs. 42.8, p < 0.001), and in particular women aged 60–65 years were almost twice as likely not to return for a second exam. Lost to follow-up women were also more likely to be born outside Spain (14.8% vs. 13.2%, p < 0.001), residing outside Barcelona (27.1% vs. 23.8%, p < 0.001), and more likely to have a negative screening result at baseline (98.9% vs. 97.0%, p = < 0.001) (Table 1).

**Table 1:** Characteristics of the study population attending screening for the first time in 2010–11 by final category at the end of the follow-up period in 2015.

	Total	Negative for HSIL+	HSIL+	*P value*
	N = 85,775	N = 85,382 (99.5%)	N = 393 (0.45%)	
**Age** mean (SD)	42.8 (10.5)	42.8 (10.5)	37.5 (9.1)	*<0*.*001*	
**Age group** N (%)				*<0*.*001*	
25–29	10,272 (12.0)	10,188 (11.9)	84 (21.4)		
30–34	11,547 (13.5)	11,462 (13.4)	85 (21.6)		
35–39	13,009 (15.2)	12,939 (15.2)	70 (17.8)		
40–44	14,349 (16.7)	14,279 (16.7)	70 (17.8)		
45–49	12,356 (14.4)	12,308 (14.4)	48 (12.2)		
50–54	9,896 (11.5)	9,883 (11.6)	13 (3.3)		
55–59	8,047 (9.4)	8,038 (9.4)	9 (2.3)		
60–65	6,299 (7.3)	6,285 (7.4)	14 (3.6)		
**Place of birth** N (%)				*0*.*058*	
Abroad	11,309 (13.2)	11,244 (13.2)	65 (16.5)		
Spain	74,466 (86.8)	74,138 (86.8)	328 (83.5)		
**Catchment area** N (%)				*0*.*004*	
Non-Barcelona	20,453 (23.8)	20,384 (23.9)	69 (17.6)		
Barcelona	65,322 (76.2)	64,998 (76.1)	324 (82.4)		
**Baseline screening category** N (%)				*<0*.*001*	
Negative	83,183 (97.0)	82,937 (97.1)	246 (62.6)		
ASC-US & HPV-	543 (0.6)	543 (0.64)	0 (0)		
ASC-US & HPV+	501 (0.6)	455 (0.53)	46 (11.7)		
ASC-US no HPV*	603 (0.7)	579 (0.68)	24 (6.1)		
LSIL	945 (1.1)	868 (1.02)	77 (19.6)		
**Time of follow-up** (months)	40.6 (12.9)	40.7 (12.9)	32.6 (18.2)	*<0*.*001*	

*HPV test not reported or inexistent within 60 days of the cervical cytology result

### Statistical analysis

Kaplan-Meier curves were used to draw the probability to develop HSIL+ over time. Curves were stratified by baseline screening results and age group: negative cytology, ASC-US HPV positive, ASC-US HPV negative, ASC-US with unknown HPV status, and LSIL and two age groups, < 35 years and 35+. Time to event was measured as person-months from the baseline cytology until an HSIL+ diagnosis or until the last cytology result, within the study period, for the remaining cytology results. Log-rank tests and the Renyi test were used to evaluate subgroup differences between cumulative incidence curves [17,18].

Multivariable logistic regression models with a complementary log-log binomial regression were used to estimate hazard ratio (HR) of HSIL+ and 95% confidence intervals (CI) [19].

Data analyses were conducted using R (R Foundation for Statistical Computing, Version 3.1.1, Vienna, Austria) and Stata (Stata 138 Statistical Software Release 15, StataCorp LLC, College Station, TX).

## Results

Table 1 describes the characteristics of the study population at baseline and of those with HSIL+ at the end of the study period. The average age of the population was 42.8 (± 10.5) years. Women aged 40–44 years was the largest age group (16.7%) while those aged 60–65 years was the smallest (7.3%). A clear majority of women were born in Spain (86.8%) and were resident in the Barcelona catchment area (76.2%). At baseline, 83,183 women (97.0%) had a negative cytology, 945 (1.1%) LSIL, and 1,647 (1.9%) ASC-US, of which 36.6% did not have a concomitant HPV test associated. Among those with an HPV test, 52% had a negative test. Average follow-up time was 40.6 months (± 12.9). During the study period, 393 women (0.5%) were diagnosed with HSIL+ that originated in the ASC-US (N = 77), in LSIL (N = 77) and in women with a negative cytology (N = 246) as baseline categories. Globally, HSIL+ cases were significantly more likely to be younger (37.5 years) and to be from the Barcelona catchment area than those with no HSIL+.

**Fig 1:**
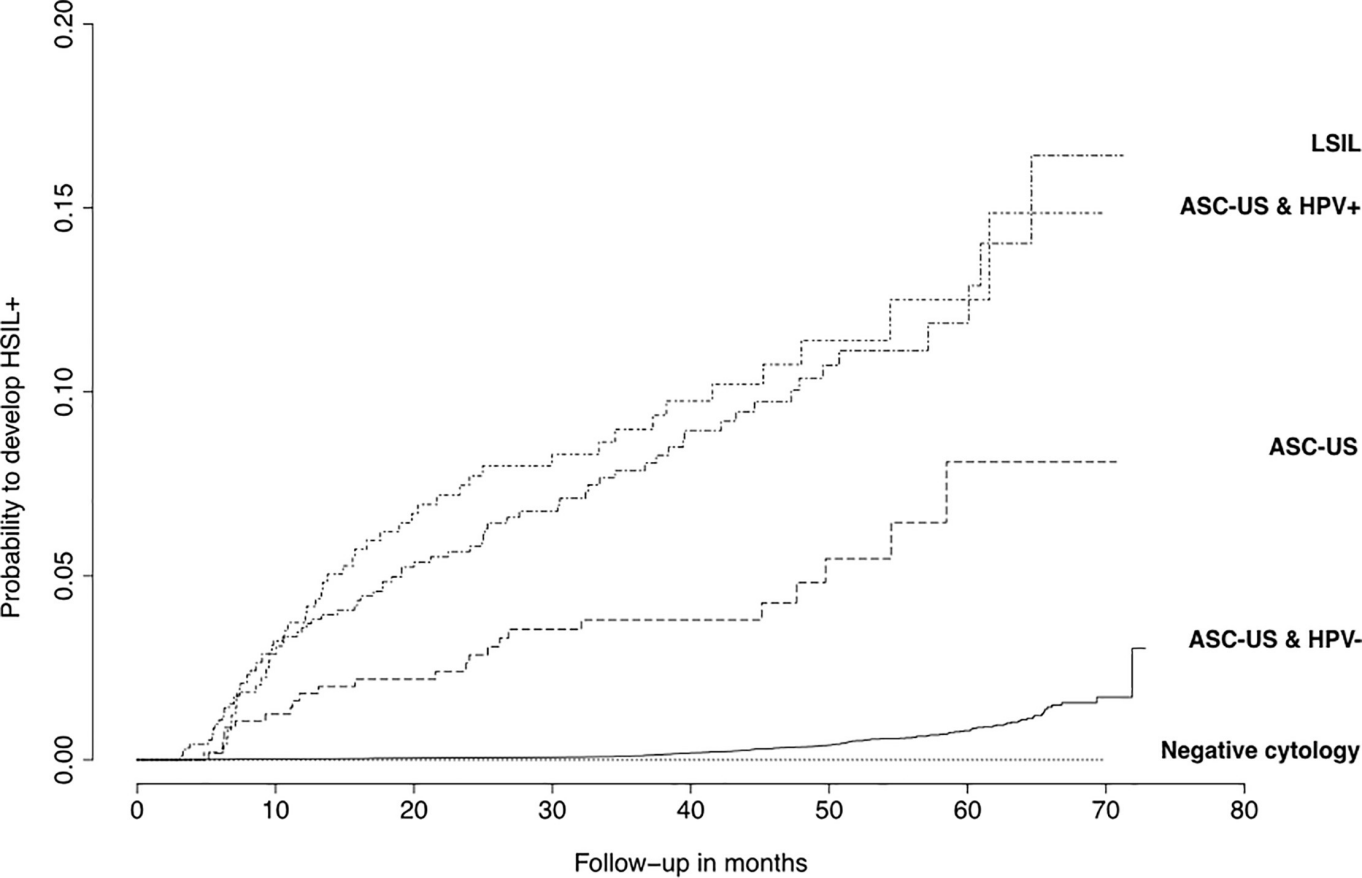
Probabilities to develop high-grade squamous intraepithelial lesion or more (HSIL+) during follow-up by age group and baseline screening diagnosis. ASC-US: atypical squamous cells of undetermined significance; HPV: human papillomavirus; LSIL: low-grade squamous intraepithelial lesion; HSIL+: high-grade squamous intraepithelial lesion or more.

Fig 1 shows Kaplan-Meier probability curves to develop HSIL+ stratified by age group and baseline screening results. The probability curve of HSIL+ among ASC-US HPV positive was statistically similar to that of LSIL (p = 0.58 among < 35 years old and p = 0.61 for those > 34 years old). Similarly, ASC-US HPV negative women and women with a negative cytology result harbored a similar probability of HSIL+ over time p = 0.50 among < 35 years old and p = 0.29 for those > 34 years old). Conversely, the probability of HSIL+ among ASC-US with unknown HPV status differed by age : younger women aged less than 35 years old had a similar detection profile of HSIL+ to that observed for ASC-US HPV positive, whereas older women aged 35 years or more presented an intermediate pattern between ASC-US HPV positive and ASC-US HPV negative.

Table 2 describes the incidence rate of HSIL+ and the HR of HSIL+ by baseline screening results adjusted by age and catchment areas. Follow-up time was slightly shorter for LSIL and ASC-US HPV positive women (34.5 and 35.3 months) compared to other categories. Women with an ASC-US HPV negative had a similar HR to women with a negative cytology (HR = 1). While women with an ASC-US HPV positive or with LSIL both had a similar increased HSIL+ risk compared to women with a negative cytology (HR = 32.7; 95% CI: 23.6–45.2; HR = 29.3; 95% CI: 22.4–38.2, respectively). Results were similar for both age groups 25–35 years and ***≥*** 35+ years with the exception of those aged ***≥*** 35+ years with an ASC-US with unknown HPV result in which the risk of HSIL was of significantly lower compared to that of HPV positive, although it remained high (HR = 19.8; 95% CI: 11.6–33.8).

**Table 2:** Hazard ratio for HSIL+ at the end of follow-up by baseline screening category.

Screening result at baseline	N	Person-years *10^5^	Mean time of follow-up (SD)	Cumulative HSIL+ cases	Incidence Rate HSIL+ %	Adjusted Hazard Ratio (95% CI)^a^
***All women***						
**Negative**	83,183	282,620.8	40.8 (± 12.7)	246	0.087	Reference
**ASC-US & HPV negative**	543	1,716.4	37.9 (± 15.8)	0	0	~1
**ASC-US & HPV positive**	501	1,474.6	35.3 (± 17.5)	46	3.1	32.7 (23.6–45.2)
**ASC-US unknown HPV**	603	1,790.4	35.6 (± 17.6)	24	1.3	14.8 (9.7–22.5)
**LSIL**	945	2,720.3	34.5 (± 18.3)	77	2.8	29.3 (22.4–38.2)
***Women 25–34 y*.*o*.**						
**Negative**	20,729	69,339.6	40.1 (± 14.3)	106	0.15	Reference
**ASC-US & HPV negative**	144	466.3	38.6 (± 16.6)	0	0	~1
**ASC-US & HPV positive**	260	760.7	35.1 (± 18.2)	19	2.5	16.5 (10.0–27.0)
**ASC-US unknown HPV**	212	636.5	36.0 (± 17.4)	9	1.4	9.4 (4.7–18.6)
**LSIL**	474	1,396.7	35.3 (± 18.5)	35	2.5	16.5 (11.2–24.3)
***Women ≥ 35 y*.*o*.**						
**Negative**	62,454	213,281.2	41 (± 12.1)	140	0.07	Reference
**ASC-US & HPV negative**	399	1,250.1	37.6 (± 15.6)	0	0	~1
**ASC-US & HPV positive**	241	713.9	35.5 (± 16.8)	27	3.8	61.5 (40.4–93.5)
ASC-US unknown HPV*	391	1,153.8	35.4 (± 17.7)	15	1.3	19.8 (11.6–33.8)
**LSIL**	471	1,323.6	33.7 (± 18.1)	42	3.2	49.3 (34.8–70.0)

SD: standard deviation; ASC-US: atypical squamous cells of undetermined significance; HPV: human papillomavirus; LSIL: low-grade squamous intraepithelial lesion; HSIL+: low-grade squamous intraepithelial lesion or more; CI: confidence interval; HPV test not reported or inexistent within 60 days of the cervical cytology result

^a^Adjusted for age and recruitment area.

*HPV test not reported or inexistent within 60 days of the cervical cytology result

Table 2 describes the detailed time related probability to HSIL+ by result of first screening cervical cytology and by age group.

## Discussion

Using a large population-based data set, the study confirms the predictive value of HPV triage in screen-detected ASC-US for HSIL+. This, to our knowledge, is the first report of these characteristics in Spain and adds to the value of molecular approaches to cervical cancer screening. The data confirm that women with a diagnosis of ASC-US HPV negative had a very low risk of HSIL+ compared to women with a negative cytology and in line with Demarco et al., supporting managing algorithms based on risk estimation [20]. Conversely, ASC-US HPV positive women had a more than 29-fold increase in risk of HSIL+ comparable to that observed among women with a cytology result of LSIL. The results are consistent with those from randomized trials [21] and observational data [22]. The data confirm that ASC-US HPV positive women have a short time to disease detection as also observed by others [23]. The fact that the risk of HSIL+ in ASCU-US HPV positive women remained high in the observation period is likely to be explained in part by delays in referral to colposcopy and getting a final diagnosis rather than a precursor lesion that has evolved during the observation window. One of the major advantages of the risk stratification is that patients should benefit from immediate referral to colposcopy, avoiding additional delays.

The similarity in risk for HSIL+ of ASC-US HPV positive and LSIL strongly suggests that management of both entities should be the same as recommended in the European Union guidelines [4]. The guidelines propose that women with ASC-US or LSIL at triage after an initial HPV primary test in a screening episode may be followed up by retesting, preferably after 6–12 months, or referred directly to colposcopy. Although risk of progression during a limited time window may be minimal [24], patient satisfaction is likely to increase by diminishing the time of the overall process from suspicion to diagnosis and treatment. We could not identify statistically significant differences between the two age categories before and after 35 years old. Therefore, the data suggest that for both ASC-US HPV positive and LSIL women, a wait and see for 24 months remains a reasonable strategy irrespective of age in the absence of a better triage test that further stratifies risk.

We had evidence of 11 invasive cervical cancers among HSIL+, 2 of them arising in the baseline category of ASC-US HPV positive result at age 26 and age 37. The time at diagnosis was 30 and 42 years old, respectively. Most likely, the total number of invasive cervical cancer cases in our series was underestimated as only those cases recorded by the general practitioner could be retrieved. This reporting is not compulsory and thus a comprehensive accountability at the primary health level cannot be guaranteed. Three out of the 11 cervical cancer cases were diagnosed in the 25–34 age group, of which 2 had as a baseline diagnosis a negative cytology and another case had an LSIL. Whether these results justify co-testing with HPV in younger women is a subject of discussion, and cost-efficiency analysis generally do not support this approach as many women would be carriers of acute infections not leading to disease. The aim of screening remains to have a test with high sensitivity followed by a good triage test. Maybe even if HPV is common in this age group, co-testing with automated cytology and HPV testing could provide a better case ascertainment than cytology alone [25,26]. To use HPV testing alone in younger women may induce too much anxiety of largely spontaneously regressing infections.

This study is based on routinely collected medical information at the primary health level. By extracting specific indicators, the data base allowed evaluation of screening practices in the absence of specific screening registries. The analysis presented informed about adhesion to general guidelines and recommendations in a routine clinical environment, covering a large population, both at geographic extent and size, and thus providing real-life estimates. However, drawbacks include information losses and disparities in data collection and clinical management over time or among health care providers. In our case, cytological records could not be comprehensively linked to colposcopy and histological confirmation results. Nevertheless, a substantial number of HSIL+ had an accompanying medical diagnosis qualifying the severity of the lesion underneath the cytology result that certified the result.

The consistency of our results with study-based projects indicate that the first step of the screening purposes, to detect HSIL+, has been clearly accomplished within the ASC-US strata particularly when HPV testing is done. It would have been desirable to obtain the full differentiation of CIN2 and CIN3+ of these lesions to evaluate potential overdiagnosis and overtreatment of doubtful cancer precursor lesions as CIN2.

The analysis of HSIL+ risk among ASC-US and HPV detection here presented has provided a strong basis to confirm the value of HPV detection for risk stratification and indicate the safety for HSIL+ risk of 5-year interval in negative results. In addition, the data presented will be relevant as baseline information and will allow evaluating future changes in cervical cancer screening policies. While HPV testing has provided an excellent tool to stratify risk in ASC-US, the possible introduction of the HPV test as a primary screening tool may result in larger improvement in risk stratification of participants to screening of cervical cancer. The possibility of setting up a robust data collection system to evaluate triage recommendations is a major achievement under the scope of opportunistic screening. We believe that these results can be of interest to countries that are re-evaluating their cervical cancer screening algorithms from using conventional cytology and moving toward introducing HPV testing.
